# Experiences of women sexual assault survivors with police in the early aftermath of assault: Results from a large-scale prospective study

**DOI:** 10.21203/rs.3.rs-4675100/v1

**Published:** 2024-07-30

**Authors:** Sara Rodríguez, Benjamin Mclean, Andrew Tungate, Andrea Massa, Jeffrey Ho, Grace Burud, Megan Lechner, Jenny Black, Jennie Buchanan, Gordon Reed, Melissa Platt, Ralph Riviello, Catherine Rossi, Israel Liberzon, Sheila Rauch, Kenneth Bollen, Samuel Mclean, Sandra Martin

**Affiliations:** University of North Carolina at Chapel Hill; University of North Carolina at Chapel Hill; University of North Carolina at Chapel Hill; University of North Carolina at Chapel Hill; Hennepin County Medical Center; University of North Carolina at Chapel Hill; University of Colorado Health; SAFE Austin; Denver Health Medical Center; Christiana Care Health System; University of Louisville Hospital; The University of Texas Health Science Center at San Antonio; Cone Health; Texas A&M University; Emory University; University of North Carolina at Chapel Hill; University of North Carolina at Chapel Hill; University of North Carolina at Chapel Hill

**Keywords:** sexual assault, posttraumatic stress, police, women’s health

## Abstract

Over 100,000 women present for emergency care after sexual assault (SA) annually in the United States. To our knowledge, no large prospective studies have assessed SA survivor experiences with police. Women SA survivors enrolled at 13 sites (*n* = 706), and 630 survivors reported on their police interactions. Most women were interested in speaking with police, spoke with police, and reported positive experiences. Latinas and women with lower education and income were less likely to speak with police. Trauma and posttraumatic stress symptoms were associated with more negative experiences. Qualitative comments provide key points for police to consider when speaking with survivors.

## BACKGROUND

More than 100,000 women in the United States (US) seek emergency medical care after sexual assault (SA) each year (1, 2). Emergency care for women SA survivors in the US is most commonly provided by a nurse with specific training and expertise in the care of these patients, termed a Sexual Assault Nurse Examiner (SANE). Forensic assessments performed by SANEs include a thorough medical and forensic history, detailed physical examination to document trauma, and evidence collection. In addition, women are also offered the opportunity to speak with police. Police evaluate SA survivors seeking emergency care to assess survivor safety and survivor willingness to file an official record of the assault (i.e., a police report).

Police interactions with SA survivors are potentially challenging. Police traditionally obtain information by functioning as impartial, dispassionate fact collectors, whereas survivors may be profoundly traumatized, vulnerable, distressed, and in need of validation and support to avoid further traumatization. Consistent with these challenges, a number of studies have reported high rates of dissatisfaction with police among SA survivors (3, 4). However, most of these studies recruited relatively small numbers of SA survivors from the general community months or years after assault and the degree to which these findings generalize to the broader SA population is not known. To our knowledge, no large prospective multisite studies recruiting SA survivors at the time of SANE care have assessed survivors’ reporting to, and experiences with, police. Obtaining a deeper understanding of SA survivors’ experiences with police, as well as factors that contribute to more positive or negative evaluations of police interactions, could inform initiatives to improve policing practices.

In this study we evaluated SA survivor satisfaction with police, using quantitative and qualitative data obtained from a large cohort of SA survivors approached at the time of SANE exam for study enrollment. Because enrolled survivors had elected to have a SANE exam with evidence collection, we hypothesized that most women would want to file a police report. In addition, based on previous evidence (5, 6) we hypothesized that socioeconomically disadvantaged groups would be less likely to speak to police and file a report. We also explored associations between trauma history and posttraumatic stress (PTS) symptoms and experiences with police, and we reviewed qualitative comments from a single open-ended question regarding what police could have done better.

## METHODS

### Study Design

The current study is based on analyses of a large-scale observational study (details omitted for blind review) of women presenting to emergency care following SA. Methods have been described in detail elsewhere (7). Briefly, adult women SA survivors presenting for emergency care at one of thirteen participating sites in a national sexual assault research network from 2015 to 2019 were recruited for the current study. Inclusion criteria included being at least 18 years of age and presenting for emergency care within 72 hours of SA. Exclusion criteria included: inability to provide informed consent, pregnancy, planning to live with the assailant after the assault, fracture, hospital admission, not speaking or reading English, no telephone access, no mailing address, unwilling to provide blood sample, incarceration, and in the opinion of study staff, unable to follow the study protocol. Men were excluded because women comprise the vast majority of SA survivors presenting for emergency care (8, 9, 10), gender differences in the development of SA sequelae exist (11), and the small male sample that could be recruited would be insufficient for stratified analyses.

### Protocol

**Figure 1:**
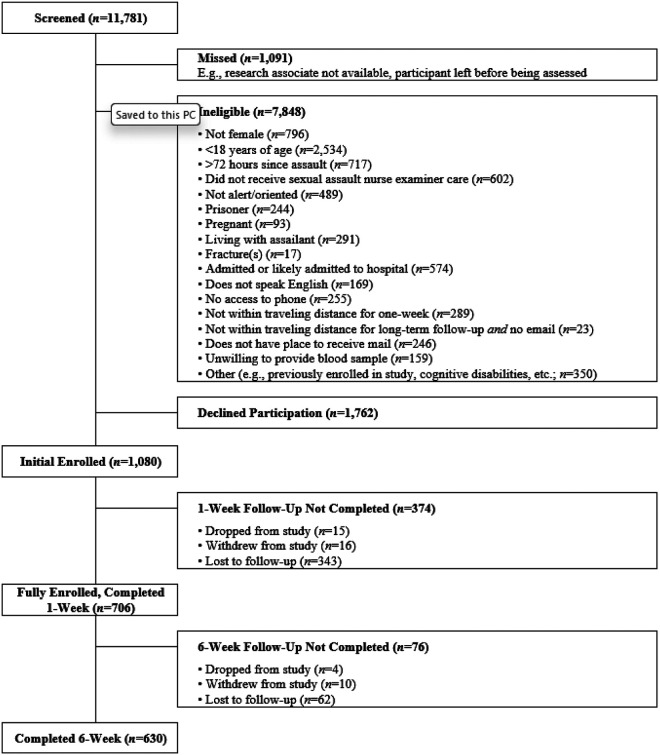
Depiction of study flow

Individuals were approached at the time of emergency care to determine their eligibility and willingness to provide initial information and learn about the study. Interested individuals provided written informed consent to share medical records and to be contacted by phone within 24–48 hours to learn more about the study. Women who were contacted and willing to participate provided full written informed consent at one week and completed assessments at one and six weeks (all one-week assessments were completed in person via laptop computer, while six-week assessments could be completed in person or via internet or phone to accommodate those who could not attend in person; Fig. 1). Participants were compensated $20 for the initial assessment and $25–50 for the six-week time point for their time and participation. All procedures were approved by Primary Investigator’s (PI’s) Institutional Review Board, as well as the local Institutional Review Board for each site.

### Demographics

Demographic data including age, race, ethnicity, work status, educational attainment, and income were collected at one week. Mutually exclusive racial categories were defined as follows: individuals who self-identified as Native Americans (with or without other racial categories) were categorized as Native American. Participants who self-identified as Black were categorized as Black, unless they also self-identified as Native American. Participants who identified as Asian, or Asian and “Other” or Pacific Islander, were categorized as Asian. Participants who identified as White and no other racial category were identified as White. Participants who identified as White and any other racial group were categorized according to the other group. Participants who identified as Pacific Islander and “Other” were categorized as “Other.”

### Assault Characteristics

Assault characteristics were obtained from the SA medical/forensic records. These records were coded by trained undergraduate research assistants, and post-baccalaureate and doctoral research staff, under supervision of the PI. Training included review of the coding protocol and practice coding of four records, which were then reviewed to assess reliability. Coding was regularly assessed for accuracy, and each record was double coded by separate research assistants.

### Measures

#### Lifetime Trauma History.

Participants completed a 15-item self-report assessment evaluating trauma exposure prior to the SA (e.g., previous assault, natural disaster, serious accident; (12). Total scores were created by summing whether or not participants had experienced each trauma type.

#### Adverse Childhood Experiences Scale (ACES).

The ACES is a 10-item self-report measure of whether participants experienced various childhood traumatic experiences (13). ACES responses were sum scored and used as an additional more specific adjuster for trauma exposure during childhood.

#### Patient-Reported Outcomes Measurement Information System (PROMIS, (14).

PROMIS forms 8a and 8b were used to measure self-reported anxiety and depression symptoms at six weeks, respectively. Each PROMIS form contains eight items rated on a five-point Likert scale (1 = *Never* through 5 = *Always*). The PROMIS forms have demonstrated excellent psychometric properties, including internal consistency, construct validity, and discrimination of clinically significant symptoms (14). A t-score of > 60 was used to classify those with significant symptoms (14).

#### PTSD Checklist – 5 (PCL-5; (15, 16).

The PCL-5, a 20-item self-report measure of PTS, was administered at one- and six-week follow-up. Participants rated current DSM-5 PTS symptoms (e.g., *Repeated, disturbing dreams of the stressful experience)* on a 4-point Likert scale (0 = *Not at all*; 4 = *extremely*). Instructions were modified to ask participants to rate symptoms related to their recent SA. The PCL-5 has excellent psychometric properties, including internal consistency, test-retest reliability, and convergent and discriminant validity (17). A score of > 33 was used to define substantial PTS symptoms (18).

#### Pain Severity Numeric Rating Scale (NRS, (19).

Pain severity (0–10 NRS score) at six weeks was assessed in each body region using an adapted version of the Regional Pain Scale (RPS; (20). Pain was described as “physical pain,” including “pain/tenderness” and “pain or aching,” to specifically assess physical, rather than emotional, pain. Pain severity in each body region during the week prior to assault was assessed at one week evaluation using the same methodology. Change in pain score of ≥ 2 in one or more body regions was defined as clinically significant new or worsening pain (19).

#### Experiences with police.

The six-week follow-up assessment included an evaluation of whether the survivor was interested in speaking with the police, and if they spoke with the police, when they did so. In addition, among women who spoke with the police, brief survey items assessed their experiences with the police, followed by the open-ended question, *“is there anything that the police could have done better?”*

### Data Analyses

Descriptive analyses were used to assess participant and assault characteristics, initial treatment at the time of emergency care, the prevalence of contacting the police, survivor experiences with the police, and survivor demographic and clinical characteristics (Tables 2 and 3). Student’s *t*-tests (two-tailed) were used to compare PTS symptoms associated with police experiences. Analyses were performed using SPSS Version 25 and *R*(21, 22). The influence of loss to follow-up was assessed by evaluating demographic or clinical differences between participants who did and did not complete 6-week follow-up assessments. All participants with 6-week follow-up data were included in analyses (Fig. 1). A qualitative thematic approach was applied to item responses to identify common themes ((23); (24). Specifically, S.R. reviewed all responses, identified themes, and categorized comments as positive or negative. Of note, individual responses could contain multiple comments regarding one or more themes, both positive and negative. A second author (S.L.M.) then reviewed comments and any discrepancies were adjudicated by the authors to determine final categorizations.

## RESULTS

### Participant Characteristics

Adult women SA survivors were screened for eligibility at the time of emergency care. Among eligible survivors who were approached (2,842), 1,080 provided initial consent at the time of emergency care, and 706 were enrolled into the full study (Fig. 1). Most enrolled SA survivors were white, less than 30 years of age, and had some post-high school education (Table 1). More than 1 in 4 survivors were Latina (181/706 [26%]). The median annual participant income was $20,000-$39,000. Childhood trauma burden was high (ACES mean score 3.83 [SD 2.85]), as was burden of lifetime trauma (mean lifetime trauma history score 5.41 [SD 3.62]). PTS symptoms in the early aftermath of SA were also high: at one week and six weeks, 530/670 (79%) and 377/613 (62%) women met criteria for substantial PTS symptoms, respectively. Among enrolled SA survivors, 630/706 (89%) completed 6-week follow-up evaluations assessing experiences with police and comprised the study sample. No significant differences in demographic or clinical characteristics were observed between SA survivors who did and did not complete 6-week follow-up evaluations (Table 1).

### Assault Characteristics

Among women who were conscious throughout the assault (286/705 [41%]), penile-vaginal penetration was reported by 248/279 (89%). Assault by a stranger was reported by 63/277 (23%), strangulation during assault was reported by 77/241 (32%), and assault by multiple individuals was reported by 17/285 (6%).

### Sexual assault survivor willingness/interest in speaking with police

Most women SA survivors were interested in speaking to the police (470/628 [75%]). Among women who were not interested in speaking with the police (158/630 [25%]), the most common reasons given were, ‘I was too upset or embarrassed’ (25%), ‘I was not sure if I had been assaulted’, or ‘I was confused about what had happened’ (17%), and ‘the police have not been helpful to me in the past’ (13%). The great majority of women who spoke to the police filed a report or made a statement (385/411 [94%]). Median number of days after assault that police spoke with the survivor was 1 (range 0–60). The great majority of women spoke to police within a week of assault (330/388 [85%]).

### Sociodemographic factors associated with not speaking with the police

Women SA survivors with a lower education level were less likely to speak with the police (*t* = 3.18, p = .00155). For example, 121/198 (61%) of women with a high school education or less spoke to the police, versus 298/428 (70%) of women with some college education. Similarly, 129/225 (57%) of women with an annual income of ≤ $20,000/year spoke to police, versus 254/358 (71%) of women with an income of > $20,000/year (*χ*^2^ = 11.37, p = .0008). In addition, only 95/163 (58%) of Latinas spoke with the police versus 307/453 (68%) of non-Latinas (*χ*^2^ = 4.76, p = .0370). None of these groups reported less interest in speaking with the police (data not shown). There were no significant differences in police contact between Black and White women.

### Experiences of sexual assault survivors who spoke with the police

The great majority of women who spoke with the police were satisfied with the interaction (Table 2). The majority of women were satisfied with how the officer treated them (344/402 [86%]) and agreed or strongly agreed that the officer treated them with respect (371/406 [91%]), believed them (357/399 [90%]), listened to them (384/409 [94%]), and did not blame them (366/402 [91%]). The great majority also agreed or strongly agreed that the officer had taken their needs and concerns seriously (358/408 [88%]), asked if they had any concerns about their safety (345/407 [85%]), asked if they had any questions (388/409 [95%]), explained the next steps in the investigation (338/409 [83%]), and explained how to contact the police for further help or information (389/410 [95%]).

### Trauma and symptom characteristics associated with police experiences

Exploratory analyses evaluated associations between survivor lifetime trauma burden and current PTS symptom burden and experiences with police. Women SA survivors with a greater burden of adverse childhood events were less likely to agree/strongly agree that police believed them (*t*=−2.43, *p* = .019) or treated them with respect (*t*=−2.39, *p* = .023). Similarly, survivors with a greater burden of previous life trauma were also less likely to agree/strongly agree that police believed them (*t*=−2.47, *p* = .0017) or treated them with respect (*t*=−3.43, *p* = .0016) and were less likely to agree/strongly agree that they were satisfied overall with how the officer treated them (*t*=−2.63, *p*= .011). Survivors with greater PTS symptoms also had significantly worse overall satisfaction with the police (*t*=−2.17, *p* = .034). There were no differences in experiences with police between Black and White survivors.

### Qualitative findings

Among women who spoke to the police, 359/411 (87%) responded to the open-ended question, *“Is there anything that the police could have done better?”* Analyses of responses identified three major themes. Example comments from each theme are described below; a complete listing of comments within each theme is provided in Supplementary Table 1.

#### Respect/Emotional Support.

Positive comments within this theme reflected the satisfaction of the great majority of survivors with police interactions. Representative positive comments included “they were very thorough and respectful,” and “I was treated with respect and care.” Negative comments underscored the central importance to survivors of being supported and treated with respect, and the additional distress caused when survivors felt judged. Examples of such negative comments included, “…quit looking at me like I had done something wrong;” “…take the situation seriously instead of like I am a liar,” and “could have been less condescending and treated me like the rape wasn’t my fault.” Another survivor stated, “they basically make you feel stupid, and ultimately at fault.” Another thread of negative comments within the *Respect/Emotional Support* theme was made by survivors who felt that they endured additional distress because police did not manage interactions in a manner that recognized their acutely traumatized state. Examples of such criticisms included that police used too many officers, made changes in officers, used male rather than female officers, used uniformed officers, used physically imposing officers, and stood in the room with the survivor in a location that made the survivor feel trapped and cornered. For example, one survivor stated, “My officer unintentionally intimidated me with his size, reminding very much physically of the person who attacked me. This was further exacerbated by sitting between myself and the door. Even though I had a female advocate in the room with me, I felt trapped…I would have felt more empowered if he did not sit between myself and the door.”

#### Communication.

Representative positive comments within this theme included, “the officer was very nice and listened to me,” “the officers did a great job of answering all of my questions and making sure I had their contact information,” and “I believe that I was…given a good deal of information encouraging me to make a report.” Representative negative comments included, “they could have explained things (especially the process/procedures/my rights) more thoroughly,” “the officers did not stop asking questions when asked to stop,” and “the officers were cold and lacked empathy during communication, and did not explain things thoroughly.” One negative comment related to breach of confidentiality, i.e., “I asked them to be discreet and they sent mail to my home with sex crime on the envelope which caused my family to question me.”

#### General/Overall Job Performance.

Most comments in this category were positive, as negative comments tended to be about specific actions. Examples of positive comments included, “I thought they did a great job,” “she was very thorough, kind, and made me feel comfortable about everything,” and “they were wonderful.” The only two negative general comments were “everything” and “she could have done a lot better.”

## DISCUSSION

To our knowledge, this is the first-in-kind large-scale prospective study surveying more than 600 adult women SA survivors six weeks after they received a SANE exam with evidence collection. Women were recruited from twelve SA emergency care sites in eleven states. Six weeks after assault, more than three quarters of women continued to experience clinically significant PTS, anxiety, or depressive symptoms. Most reported speaking with the police and filed a statement or report. Experiences with law enforcement among women who spoke with the police were overwhelmingly positive. Nearly 9 out of 10 women were satisfied overall with how they were treated and more than 9 out of 10 women felt that the police officer believed them and treated them with respect. Experiences with police did not differ by race or ethnicity. However, individuals with a greater burden of childhood or lifetime trauma were less likely to agree that police treated them with respect or believed their story. Women with a higher burden of PTS were also less satisfied with their treatment by police. Police contact rates were significantly lower among Latinas and women with a high school education or less and those with an income of less than $20,000/year. The most common reasons for not contacting the police were embarrassment, fear of the assailant, and uncertainty regarding what happened during the assault.

Most studies to date that have examined SA survivor experiences with police have performed qualitative analyses of interviews with relatively small samples of survivors recruited from the community via flyers and advertisements (3, 25, 26). The most common themes of women with negative police experiences in these studies, not feeling believed and not feeling treated with respect, are consistent with the most common themes of women in the present study who reported negative experiences. These findings are consistent with the fact that women with a high burden of PTS and those with greater lifetime burden of trauma exposure were less likely to be satisfied with their police interactions. These data highlight the importance of police alertness and sensitivity to the profound sense of vulnerability of survivors in the early aftermath of assault. Body language, room position, and tone of voice that would not normally be problematic can cause distress and suffering among individuals experiencing intense fear responses in the early aftermath of severe traumatic stress. The findings that survivors with a greater burden of PTS symptoms and survivors with a greater burden of previous life trauma (who also had more acute stress symptoms) reported more negative experiences likely reflects that high quality police work among individuals in the immediate aftermath of extreme trauma requires a high level of consideration for such potential feelings of vulnerability.

The above-described community-based studies, and analyses of our qualitative data, are valuable for identifying common causes of negative interactions that increase survivor burden. However, because such studies have not more systematically assessed police interactions of a cohort of SA survivors, they do not provide information regarding the frequency with which negative police interactions occur. To our knowledge, our study is the first large scale prospective study to evaluate experiences with police among a cohort of adult women SA survivors presenting for emergency care. Our finding that the great majority of women SA survivors were satisfied with their police interactions in the immediate aftermath of the assault is consistent with generally positive experiences with police and/or detectives reported by SA survivors in small cohort-based studies performed in other settings (27); (28). Meanwhile, the higher rate of negative qualitative comments in our study as compared to positive quantitative survey responses regarding police interaction may reflect an inherent negativity bias in open-ended questions and/or the fact that open-ended questions are particularly susceptible to non-response bias (O’Cathain and Thomas, 2004; Poncheri et al., 2008).

Latinas and women with the lowest educational opportunity and lowest income were less likely to speak with police. As noted above, the most common reasons for not contacting the police were embarrassment, fear of the assailant, and uncertainty regarding what happened during the assault. These findings highlight the importance of ongoing work to make law enforcement a supportive space for disclosure (29); (30).

Our results suggest that most SA survivors are satisfied with their interactions with police. However, there remain survivors who note difficulties, particularly those with a greater burden of trauma exposure and PTS symptoms. Given this finding, police encounters with SA survivors receiving emergency care may benefit from the consistent use of a trauma-informed approach. We propose that this approach can be summarized and recalled using the mnemonic “SABE” (“to know”). Police should demonstrate empathy and respect by beginning interactions with survivors with the statement, “I am so **S**orry that this happened to you.” Police should **A**sk permission of the survivor frequently, to ensure that the survivor feels in control of the interaction and respected. Examples of asking permission include, “Would it be OK if I spoke to you for a few minutes,” “Is it OK if I come in the room,” “Is it OK if I sit here, or would you prefer that I stand.” After the survivor provides a history, to ensure that they feel validated and believed, state “I **B**elieve you.” Finally, **E**xplain what is going to happen, both during and after the interview, and ensure that all of their questions are answered.

This study had several limitations which should be considered when interpreting our results. Only adult women presenting for emergency care after SA were included, due to the fact that they compose the vast majority of survivors seeking emergency care (2, 31). Experiences with police among other survivor populations were not assessed. Similarly, experiences with police among women SA survivors who did not present for emergency care, or did not consent to the study, are unknown. Also, this study focused on experiences with police in the early aftermath of assault, at the time of filing a police report, and not experiences with police, detectives, or the legal system at later timepoints. Finally, police contact was assessed via self-report. However, the time of recall was relatively short, and this allowed us to assess a broad range of police experiences (e.g., crisis hotline, substance abuse counselor) and assault disclosure.

## CONCLUSIONS

In conclusion, in this cohort study of more than 600 adult women SA survivors presenting for emergency care to one of 12 SANE programs in 11 states, nearly 9 out of 10 women were satisfied overall with how they were treated and more than 9 out of 10 felt that the police officer believed them and treated them with respect. Latinas and women with lower education and income were less likely to speak with the police. Women with a high burden of PTS symptoms and those with a greater lifetime trauma burden were less likely to have positive police interactions. These data highlight the importance of police alertness and sensitivity to the profound sense of vulnerability of survivors in the early aftermath of assault. Potential pitfalls can be avoided using the mnemonic “SABE” (“know”): (1) demonstrate empathy and validation via statement “I am so **S**orry that this happened to you;” (2) **A**sk permission of the survivor frequently during the interaction, to ensure they feel in control; (3) State “I **B**elieve you” to provide validation after survivor provides a history, and (4) **E**xplain what is going to happen, both during the interview and going forward, and ensure that all of their questions are answered. The use of such standardized methods can help ensure that the very best policing practices are provided to crime victims at a time of tremendous vulnerability.

## Figures and Tables

**Table 1:** Characteristics of adult women sexual assault survivors who were enrolled in the full study (n = 706) and who completed six-week follow-up evaluation assessing experiences with police.

	Enrolled (*n* = 706)	Completed six-week follow-up (*n* = 630)	*p* Value
Age, *M* (SD)	28.4 (9.7)	28.4 (9.7)	.973
Race, n (%)			.904
White	397 (57.3%)	355 (57.2%)	
Black	104 (15.0%)	88 (14.2%)	
Asian	13 (1.9%)	10 (1.6%)	
Native American	76 (11.0%)	74 (11.9%)	
Other	103 (14.9%)	93 (15.0%)	
Latina Ethnicity, n (%)	181 (26.3%)	164 (26.6%)	.855
Education, n (%)			.993
Less than high school	56 (8.0%)	50 (8.0%)	
High school or equivalent	172 (24.6%)	149 (23.8%)	
Post-high school/some college	330 (47.1%)	298 (47.6%)	
College degree	113 (16.1%)	103 (16.5%)	
Graduate degree	29 (4.1%)	26 (4.2%)	
Annual Income, n (%)			.994
<$20,000	254 (38.9%)	225 (38.6%)	
$20,000–39,999	156 (23.9%)	140 (24.0%)	
$40,000–79,999	159 (24.4%)	145 (24.9%)	
$80,000+	83 (12.7%)	73 (12.5%)	
Work Status, n (%)			.965
Student	152 (22.0%)	137 (22.2%)	
Not currently working	192 (27.7%)	169 (27.3%)	
Part-time	84 (12.1%)	79 (12.7%)	
Full-time	264 (38.2%)	235 (37.8%)	

**Table 2:** Experiences of women who spoke to the police after sexual assault (n = 411, median days after assault that spoke to police = 1, range 0–60 days).

Item	Strongly Agree	Agree	Disagree	Strongly Disagree
Satisfied overall with how officer treated me	216 (54%)	128 (32%)	35 (9%)	23 (6%)
Police officer treated me with respect	257 (63%)	114 (28%)	23 (6%)	12 (3%)
Police officer believed me	223 (56%)	134 (34%)	30 (8%)	12 (3%)
Police officer did not blame me	239 (60%)	127 (32%)	26 (7%)	10 (3%)
Police officer listened to me	257 (63%)	127 (31%)	21 (5%)	4 (1%)
Police officer took my needs and concerns seriously	247 (61%)	111 (27%)	33 (8%)	17 (4%)
Police officer asked if I had any concerns about my safety	222 (55%)	123 (30%)	43 (11%)	19 (5%)
Police officer explained what was going to happen next in the reporting, investigation, or prosecution	192 (47%)	146 (36%)	49 (12%)	22 (5%)
Police officer asked if I had any questions	245 (60%)	143 (35%)	14 (3%)	7 (2%)
Police officer explained how I could contact the police if I needed further help or information	241 (59%)	148 (36%)	12 (30%)	9 (2%)

## Data Availability

A version of the de-identified database used in the analysis of this study is available upon request.

## Abbreviations

SA: Sexual Assault
SANE: Sexual Assault Nurse Examiner
PTS: posttraumatic stress
PI: Primary Investigator
ACES: Adverse Childhood Experiences Scale
PROMIS: Patient-Reported Outcomes Measurement Information System
PCL: 5-PTSD Checklist-5
NRS: Pain Severity Numeric Rating Scale
